# Application of metagenomic next-generation sequencing in the diagnosis and treatment of recurrent urinary tract infection in kidney transplant recipients

**DOI:** 10.3389/fpubh.2022.901549

**Published:** 2022-08-22

**Authors:** Wenjing Duan, Yongguang Yang, Jingge Zhao, Tianzhong Yan, Xiangyong Tian

**Affiliations:** ^1^Department of the Clinical Research Center, Henan Provincial People's Hospital, Zhengzhou University People's Hospital, Henan University People's Hospital, Zhengzhou, China; ^2^Department of Urology, Henan Provincial People's Hospital, Zhengzhou University People's Hospital, Henan University People's Hospital, Zhengzhou, China

**Keywords:** metagenomic next-generation sequencing (mNGS), kidney transplantation, recurrent urinary tract infection (RUTI), uropathogens, anti-infective treatment

## Abstract

**Background:**

Rapid and accurate pathogen diagnosis is an urgent unmet clinical need for recurrent urinary tract infection (RUTI) in kidney transplant recipients (KTRs). Metagenomic next-generation sequencing (mNGS) may offer another strategy for diagnosing uropathogens but remains to be studied.

**Methods:**

Nineteen KTRs with RUTI were collected in this study. The uropathogens were detected and compared by mNGS and urine culture, respectively. Modifications of the anti-infection strategy were also assessed.

**Results:**

Rich and diverse pathogens were revealed by mNGS. mNGS was significantly higher than culture in total positive rate (100.0% vs. 31.6%; *p* < 0.01) and in identification rates for bacteria (89.5% vs. 31.6%; *p* < 0.01), for viruses (57.9% vs. 0; *p* < 0.01), and for fungi (42.1% vs. 0; *p* < 0.01), respectively. mNGS identified a significantly higher proportion of mixed infections than culture (89.5% vs. 10.5%; *p* < 0.01). The anti-infection therapies were adjusted in two (33.3%) and 12 (76.9%) cases guided by culture and mNGS, respectively.

**Conclusion:**

mNGS has more remarkable etiological diagnostic performance compared with urine culture for KTRs with RUTI to guide anti-infection strategies and, in turn, protect the graft.

## Introduction

Kidney transplant confers profound survival benefits for the treatment of end-stage kidney disease. With the widespread application of potent immunosuppressive agents and improved organ preservation techniques recently, the 1-year survival rate of kidney transplants has increased to more than 90% (1). However, with the long-term use of large amounts of immunosuppressants to prevent rejection of the allograft, the immune function of kidney transplant recipients (KTRs) is significantly reduced, increasing the threat of postoperative infection. Recurrent urinary tract infection (RUTI) is defined as ≥3 uncomplicated urinary tract infections within 12 months or 2 uncomplicated, symptomatic urinary tract infections within 6 months (2). The periodic RUTI may lead to high medical costs, low quality of life, significant drug resistance, and in some cases, extreme renal allograft injury, and even kidney failure in KTRs, which constitutes a growing health challenge (3–5). Considering the adverse consequences of RUTI, it is of considerable importance to identify the uropathogens as quickly and as accurately as possible to devise precision therapy and preventive strategies.

Currently, conventional urine culture is recommended as an economical and convenient method to detect uropathogens for RUTI in clinical testing and can also be used for drug susceptibility testing (DST). However, this method is time consuming, prone to contamination, and difficult to identify multiple infections, and its sensitivity and specificity are unsatisfactory. Therefore, there is an urgent need to develop rapid and reliable diagnostic techniques for RUTI pathogens to address this problem in clinical practice.

Metagenomic next-generation sequencing (mNGS) is an emerging method for the identification of pathogens. Since its successful use in 2008 for detecting new pathogenic infections (6), mNGS has gradually transitioned from laboratory to clinical applications (7). This culture-independent technique allows for rapid and accurate sequence detection of pathogenic microorganisms (such as bacteria, fungi, and viruses.) without bias by directly targeting nucleic acids in clinical samples (8). A few studies have showed the potential value of mNGS for rapid diagnosis of urinary tract infections (9–11). However, for patients with clinical recurrence, it is of interest. It remains to be studied whether the uropathogens are consistent with the results of these studies, thus contributing to the understanding of recurrent infection in special populations of immunosuppressed KTRs as either relapse with the primary infecting strain or reinfection with a new strain.

This study aimed to evaluate and compare the diagnostic performance of these two methods: urine culture and mNGS, for the rapid and accurate identification of pathogens in KTRs with RUTI.

## Materials and methods

### Study design and population

**Figure 1:**
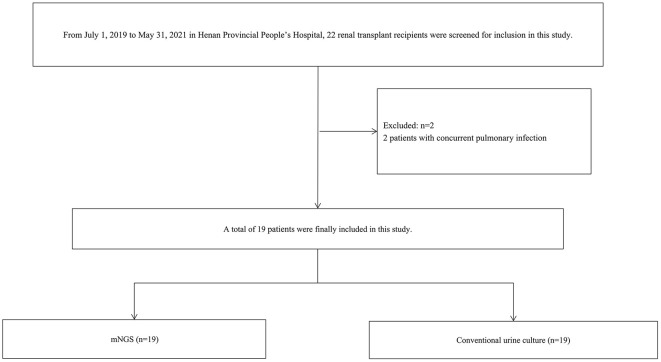
An overview flowchart showing patient enrollment.

This study was conducted in Henan Provincial People's Hospital, a tertiary teaching hospital in Zhengzhou, China, from July 2019 to May 2021. A total of 19 KTRs diagnosed with post-transplant RUTI were screened and eventually investigated in the present study (Figure 1). The inclusion criteria were as follows: (1) KTRs ≥ 18 years of age and (2) patients diagnosed with post-transplant RUTI (at least two episodes of infection in 6 months or at least three episodes of infection in 1 year). The exclusion criteria were as follows: (1) patients diagnosed with RUTI before kidney transplantation and (2) patients reporting simultaneous infections at other sites. Abstractions of patients' demographic and clinical information from the electronic medical record were collected, including age, sex, application of immunosuppression, infection signs and symptoms, laboratory examinations, risk factors of RUTI, antibiotic treatments, and the response during 6 months of follow-up. Two clinicians performed the diagnosis of RUTI. Urine samples were collected from the patients and tested by mNGS and urine culture. At least two clinicians reviewed the results to discriminate infection from contamination. Targeted adjustment of antibiotic treatment was defined as the adjustment of antibiotic treatment by the clinician according to the results of mNGS or culture, including de-escalation and escalation of antibiotics. All clinical treatments were performed based on the physician's assessment of patient symptoms and examination results.

### Metagenomic next-generation sequencing and data analysis

#### Sample collection

The collection of all samples followed the standard operating procedures conforming to the rules of the aseptic technique and was transported to the sequencing laboratory by the cold chain in time.

#### Nucleic acid extraction

According to the manufacturer's operational guidebook, TIANamp Micro DNA Kit (DP316, TIANGEN Biotech, Beijing, China) was used for DNA extraction. DNA extraction was conducted for each sample, while RNA extraction and reverse transcription were applied according to the patient's manifestations at the discretion of the physician's clinical decisions, particularly if a viral infection was suspected.

#### Library construction and sequencing

The total DNA or cDNA was subjected to library construction through an end-repair method. A specific tag sequence was introduced at the end of each library. The library concentration was determined by Qubit 4.0 nucleic acid fluorescence quantitative analyzer (Q33226, Thermo Fisher, USA) and Qubit^®^ dsDNA HS Assay Kit (Q32854, Thermo Fisher, USA). Agilent 2100 Bioanalyzer (G2939BA, Agilent, USA) was used to evaluate the DNA concentration and fragment size in the library to be sequenced for the quality control of the DNA libraries. DNA nanospheres were prepared by one-step DNB kit (1000025076, Huada Zhizao, China). The MGISEQ-200 platform sequenced quality-qualified libraries.

#### Bioinformatic analysis

After removing low-quality (<35 bp) and low-complexity reads according to PRINSEQ (version 0.20.4) and computational subtracting human host sequences mapped to the human reference genome (hg38) from the sequencing data by Burrows–Wheeler Alignment (0.7.10-r789), high-quality sequences were generated. The remaining non-host sequences were matched and classified with four self-constructed genome databases of pathogenic microorganisms consisting of bacteria, fungi, parasites, and viruses, which were downloaded from the National Center for Biotechnology Information (ftp://ftp.ncbi.nlm.nih.gov/genomes/) and other public databases.

### Conventional urine culture

Clean-catch midstream urine samples were collected, placed into BD Vacutainer Plus C&S preservative tubes, and sent to the clinical microbiology laboratory, Henan Provincial People's Hospital, within 1 h after collection for processing and analysis. Five percent of sheep blood agar plate (BAP) and MacConkey agar plate (BD BBL prepared plated media) were used for culture of bacteria at 37°C under aerobic, microaerophilic, or anaerobic conditions for 24 h. If pinpoint growth was seen at 24 h, the agars were held for another 24 h under the same conditions. Each distinct colony morphology was subcultured at 48 h to obtain pure culture for microbial identification. The fungi were cultured on Sabouraud dextrose agar plates at 37 or 27°C for 1–5 days. A positive urine culture is defined as ≥10,000 colony-forming units of a potential uropathogenic per mL of urine (12). All other urine culture results were defined as negative. Isolates were identified by matrix-assisted laser desorption/ionization time-of-flight mass spectrometry (MALDI–TOF MS; Bruker, Germany).

### Statistical analysis

Continuous data conforming to a normal distribution were reported as the mean ± standard deviation value; continuous data outside the normal distribution were presented as median and interquartile range (IQR). Categorical data were presented as the number of cases and percentage (%). The McNemar Chi-square test was conducted to compare the results of mNGS and traditional culture to determine the differences. Fisher's exact probability test was used to compare the proportion of patients with negative and positive cultures who changed the antibiotic treatment based on the mNGS results. Our data, prior literature, and power calculation determined sample sizes. Data analyses were performed using Statistical Package for the Social Sciences (SPSS) version 24.0 statistical software (IBM SPSS, Chicago, IL, USA). All P-values were two-sided, and statistical significance was defined as *P* < 0.05.

### Ethics

All kidney transplants in this study were performed in Henan Provincial People's Hospital. No organs were procured from prisoners or other institutionalized persons, and all organ donations were contributed voluntarily. Participants provided written informed consent before the collection of samples. The study was conducted following the Declaration of Helsinki. Ethical approval was provided by the Clinical Research Ethics Committee of Henan Provincial People's Hospital.

## Results

### Characteristics of the participants

Details of the clinical characteristics of the 19 KTRs with RUTI enrolled in this study are presented in Table 1. The sample size was sufficiently powered, accounting for the incidence rates and significant difference in positive rates. There were seven male and 12 female patients with an average age of 39.58 ± 11.14. Sixteen patients had a fever (body temperature >37.3°C) due to the RUTI, and all patients had lower urinary tract symptoms, including urinary frequency, urgency, pain, or burning sensation during urination, leading to impaired quality of life. Moreover, the pain or burning sensation was the most common. The following comorbidities were identified: single hypertension in 18 cases, concurrent hypertension and type II diabetes in 1 case, and ureteral stenosis causing long-term indwelling double-J ureteral stents placement in 2 cases.

**Table 1:** Baseline characteristics of participants.

**Characteristics**	**Case (n = 19)**
**Gender**	
Men	7 (36.8%)
Women	12 (63.2%)
**Age (year)**	39.58 ± 11.14^*^
**Pre-transplant dialysis durations (month)**	7 (2, 13)^#^
**Comorbidity**	
Only hypertension	18 (94.7%)
Hypertension and diabetes	1 (5.3%)
Ureteral stricture	2 (10.5%)
**Body temperature**	
Normal	3 (15.8%)
≥37.3°C	16 (84.2%)
**Symptoms of lower urinary tract**	
Frequency of urination	12 (63.2%)
Urgency of urination	5 (26.3%)
Pain or burning during urination	13 (68.4%)
**Urinalysis**	
Leukocyte characterization (1+ - 3+)	19 (100.0%)
Urine occult blood positive	6 (31.6%)
Yeast positive	1 (5.3%)
Nitrite positive	2 (10.5%)
**Serum creatinine (umol/L)**	141 (87,194)^#^
**Inflammatory indicators**	
Leukocyte (x10^9^)	7.69 (5.41, 11.94)^#^
Neutrophils (%)	83.1 (60.7, 88.7)^#^
C-reactive protein (mg/L)	29.48 (4.76, 77.51)^#^
Procalcitonin level (ng/mL)	0.35 (0.07, 1.21)^#^
**Presence of acute rejection**	6 (31.6%)
**Excessive tacrolimus blood drug concentration (>8 ng/ml)**	6 (31.6%)

* *indicates mean ± SD*;

# *indicates median (IQR)*.

All KTRs received anti-thymocyte globulin induction to prevent renal allograft rejection before surgery. After the transplant, they were initially treated with the standard immunosuppressive regimen, consisting of tacrolimus, mycophenolate mofetil (MMF), and prednisone. The tacrolimus dosage was weight-based and then adjusted according to close monitoring to maintain tacrolimus blood concentrations within the therapeutic range to ensure efficacy and safety.

### Diagnostic performance of mNGS

**Figure 2:**
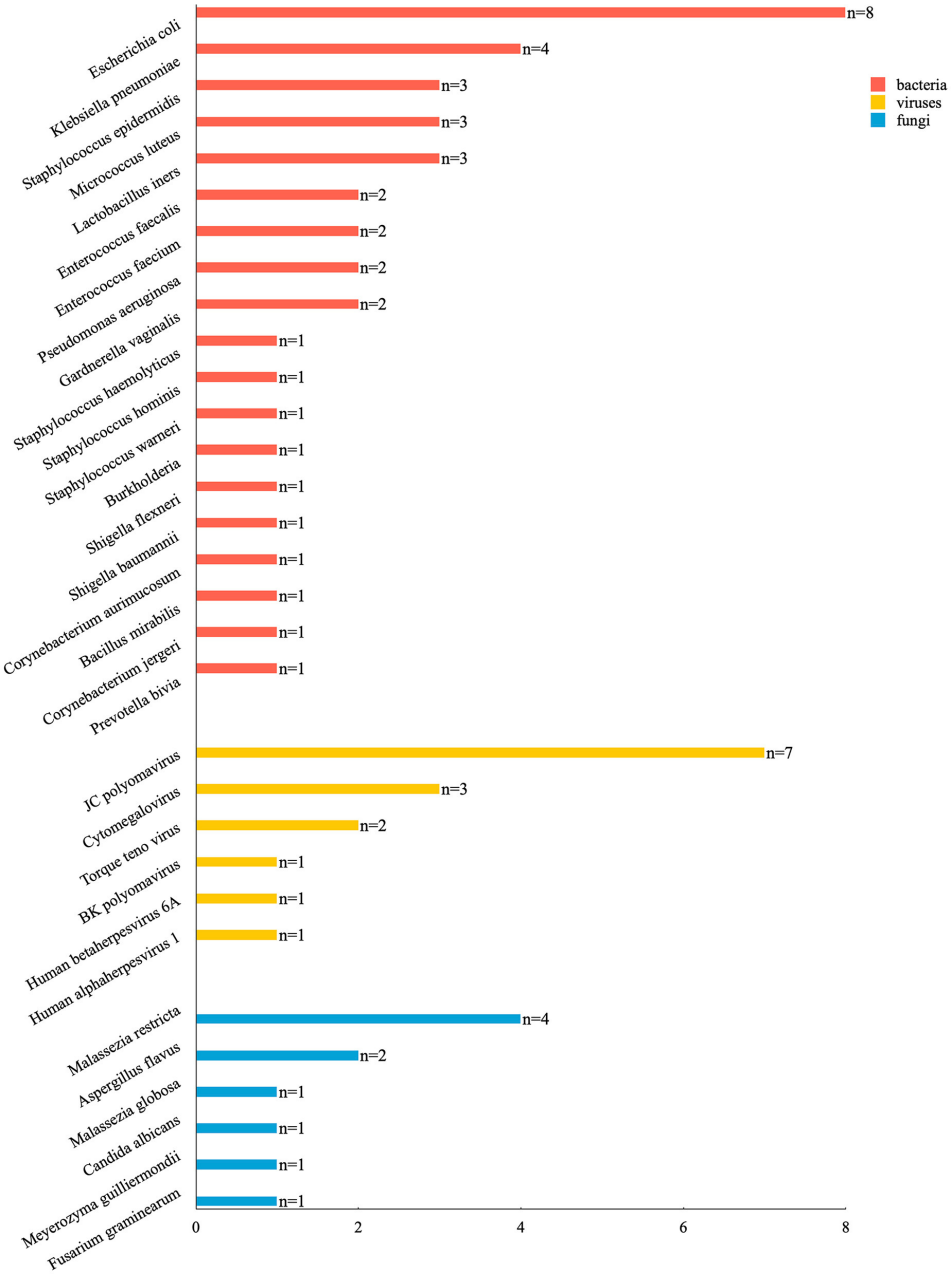
The category of pathogenic microorganisms detected by mNGS and the corresponding numbers of patients.

A total of 19 samples were tested by mNGS. A total of 19 bacterial species were detected in 17 samples (89.5%), six viruses in 11 samples (57.9%), and six fungal species in eight samples (42.1%). Table 2 and Figure 2 report the type of microorganisms. The sensitivity to detect bacteria by mNGS was 89.5% (17/19), indicating the abundance of urine bacteria. The top five were *Escherichia coli* (42.1%, 8/19), *Klebsiella pneumonia* (21.1%, 4/19), *Staphylococcus epidermidis* (15.8%, 3/19), *Micrococcus luteus* (15.8%, 3/19), and *Lactobacillus iners* (15.8%, 3/19). The positive rate of viruses was 57.9% (11/19) by mNGS, of which *JC polyomavirus* (36.8%, 7/19), *Cytomegalovirus* (CMV, 15.8%, 3/19), and *Torque ateno virus* (TTV, 10.5%, 2/19) were the most commonly seen. The positive rate of fungi was 42.1% (8/19) by mNGS, of which *Malassezia restricta* (21.1%, 4/19) and *Aspergillus flavus* (10.5%, 2/19) were the most commonly seen.

**Table 2:** The abundance and nucleic acid sequence number of various microorganisms in 19 urine samples.

**Patient**	**Bacteria [relative abundance (%); reads number]**	**Viruses [relative abundance (%); reads number]**	**Fungi [relative abundance (%); reads number]**
P1	• *Escherichia coli* (-; 22137) • *Shigella flexneri* (-; 20565) • *Shigella baumannii* (-; 20484)	-	-
P2	-	*JC polyomavirus* (-; 2012)	*Candida albicans* (-; 347)
P3	• *Corynebacterium aurimucosum* (4.21; 952) • *Staphylococcus haemolyticus* (2.19; 475)	*Torque teno virus* (98.54; 1619)	*Aspergillus flavus* (77.16; 875)
P4	• *Staphylococcus epidermidis* (75.34; 333) • *Micrococcus luteus* (13.79; 253) • *Bacillus mirabilis* (24.66; 109)	-	-
P5	*Lactobacillus iners* (100; 1976)	*Human betaherpesvirus 6A* (98.97; 385)	-
P6	*Escherichia coli* (100; 101)	• *JC polyomavirus* (90.37; 2268) • *Cytomegalovirus* (9.04; 447)	-
P7	• *Klebsiella pneumoniae* (39.12; 1262143) • *Enterococcus faecalis* (2.58; 83089) • *Escherichia coli* (0.1; 3233) • *Enterococcus faecium* (0.01; 345)	• *JC polyomavirus* (90.21; 3381) • *Torque teno virus* (2.67; 100)	-
P8	• *Micrococcus luteus* (23.13; 423) • *Klebsiella pneumoniae* (19.3; 353)	-	-
P9	• *Micrococcus luteus* (46.61; 4950) • *Staphylococcus warneri* (1.32; 140) • *Staphylococcus epidermidis* (1.05; 112)	-	*Malassezia restricta* (66.85; 121)
P10	• *Burkholderia* (30.23; 763661) • *Klebsiella pneumoniae* (0.06; 1414) • *Pseudomonas aeruginosa* (0.17; 4210)	*JC polyomavirus* (92.04; 4071)	• *Malassezia restricta* (60.43; 3727) • *Malassezia globosa* (13.77; 849)
P11	• *Lactobacillus iners* (49.78; 54121) • *Staphylococcus hominis* (1.03; 1120) • *Pseudomonas aeruginosa* (0.30; 324)	• *Human alphaherpesvirus 1* (35.14; 117) • *Cytomegalovirus* (34.23; 114)	• *Malassezia restricta* (37.97; 221) • *Aspergillus flavus* (18.04; 105)
P12	• *Lactobacillus iners* (14.35; 898) • *Escherichia coli* (5.26; 329)	• *JC polyomavirus* (88.02; 2881) • *Cytomegalovirus* (10.05; 329)	*Fusarium graminearum* (58.29; 116)
P13	*Escherichia coli* (8.73; 1621)	*JC polyomavirus* (95.87; 6830)	-
P14	-	-	*Meyerozyma guilliermondii* (79.35; 461)
P15	*Corynebacterium jergeri* (36.72; 767)	-	-
P16	• *Klebsiella pneumoniae* (41.15; 1165559) • *Staphylococcus epidermidis* (0.10; 2908) • *Escherichia coli* (0.05; 1444)	-	*Malassezia restricta* (64.02; 105)
P17	• *Gardnerella vaginalis* (45.22; 3887) • *Escherichia coli* (1.23; 106)	*JC polyomavirus* (96.72; 6832)	-
P18	• *Enterococcus faecalis* (36.28; 8319) • *Escherichia coli* (13.27; 3043) • *Enterococcus faecium* (3.57; 819)	-	-
P19	• *Gardnerella vaginalis* (73.08; 21148) • *Prevotella bivia* (2.11; 612)	*BK polyomavirus* (83.85; 2918)	-

“*-” indicates negative or missing data*.

### Diagnostic performance of urine culture

Colony growth was observed in six cases (31.6%, 6/19) by urine culture (Table 3). Species identification and DST were carried out for bacteria, indicating *Escherichia coli* (*n* = 4, 21.1%), *Enterococcus faecium* (*n* = 2, 10.5%), *Klebsiella pneumoniae* (*n* = 2, 10.5%), and *Staphylococcus epidermidis* (*n* = 1, 5.3%). No fungus was found in all urine cultures.

**Table 3:** The results of urine culture in 19 samples.

**Patient**	**Results of urine culture**
P1	*Escherichia coli*
P6	*Escherichia coli*
P7	*Klebsiella pneumoniae* + *Enterococcus faecium*
P8	*Klebsiella pneumoniae* + *Enterococcus faecium* + *Staphylococcus epidermidis*
P13	*Escherichia coli*
P17	*Escherichia coli*

### Consistency and differences between mNGS and urine culture results

**Figure 3:**
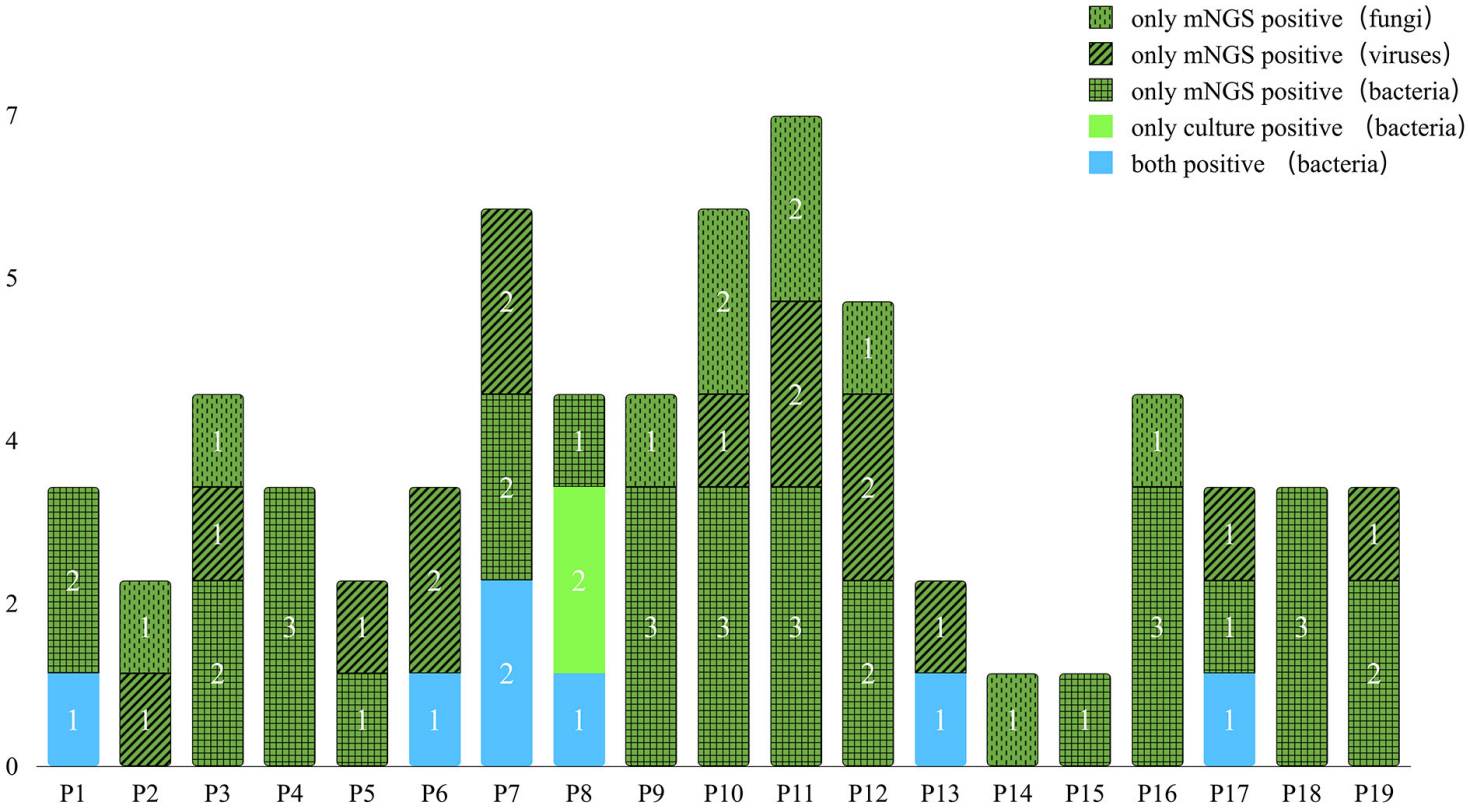
The distribution of pathogens compared by mNGS and urine culture.

All samples underwent both mNGS and urine culture methods. Figure 3 and Table 4 list the mNGS results for the 19 patients compared with the culture results. Regarding all kinds of pathogenic microbes, mNGS reached a sensitivity of 100.0%, which was much superior to the traditional culture method of 31.6%. Culture identified four kinds of bacteria in six samples, while mNGS identified 19 kinds of bacteria in 17 samples. The bacteria-positive rate of mNGS for KTRs with RUTI was significantly higher than that of culture (89.5 vs. 31.6%, *p* < 0.001). In comparison with culture, better performance of mNGS in detecting viruses and fungi (57.9 and 42.1%, respectively) was evident in these results.

**Table 4:** The positive rate between mNGS and urine culture.

	**mNGS (*n* = 19, %)**	**Urine culture**	***p*-value**
		**(*n* = 19, %)**	
Pathogens	19 (100.0)	6 (31.6)	<0.001
Bacteria	17 (89.5)	6 (31.6)	<0.001
Viruses	11 (57.9)	0	<0.001
Fungi	8 (42.1)	0	<0.001

The six positive cases found in the urine culture were a subset of the positive cases in mNGS, while the corresponding pathogens detected by the two methods were not identical. Most of the pathogens found by mNGS were not detected by urine culture. However, most of the pathogens detected by urine culture can be detected by mNGS. Nevertheless, the infection of *Enterococcus faecium* and *Staphylococcus epidermidis* was detected positive by culture but failed to be reported by mNGS in patient P8. mNGS missed two bacteria that tested positive by culture, which may be related to the patient's antibiotic treatment before mNGS. Among the 13 culture-negative cases, mNGS results were all positive, of which 11 cases were bacterial positive, seven cases virtual positive, and eight cases fungal positive. Notably, of these fungi, none were detected by culture according to the positive criteria. It is noteworthy that mNGS has tremendous advantages in detecting viruses that traditional culture methods do not, indicating mNGS' potency in detecting unexpected viruses. In addition, using mNGS, the diagnostic speed can be nearly two times faster than the traditional culture method (2–3 vs. 3–6 days).

### Comparison of the identification of infections of multiple pathogens between mNGS and urine culture

In the multipathogen infection cases, mNGS detected a mixture of bacteria in 13 cases (68.4%), a dual virus infection in four cases (21.1%), and a dual fungi infection in two cases (10.5%). Mixed infections of bacteria and viruses were detected in six cases (31.6%). Mixed fungal and bacterial infections were detected in two cases (10.5%). Mixed infection of viruses and fungi was detected in one case (5.3%). Mixed infections of bacteria, fungi, and viruses were detected in four cases (21.1%). By comparison, mNGS showed a significantly higher proportion of multipathogen infections identified than culture (89.5%, 17/19 vs. 10.5%, 2/19; *p* < 0.001).

### Antibiotic resistance gene detection by mNGS

Genes encoding antimicrobial resistance were determined from bacterial genome sequences using mNGS. Antibiotic resistance genes were detected in five (5/19, 26.3%) patients. Three kinds of bacteria are involved: *Klebsiella pneumonia, Escherichia coli*, and *Enterococcus faecalis*. The results revealed the presence of several antibiotic resistance genes, including SHV-160, mphA, CTX-M-117, APH(4)-Ia, CTX-M-50, KPC-12, SHV-110, AAC(6')-Ib7, mdtN, mdtF, efrB, and tet(C), which cause resistance to cephalosporins, carbapenems, macrolides, aminoglycoside, tetracyclines, or nucleoside antibiotics.

### Changing antibiotic treatment according to mNGS results

All patients were empirically given antibiotics for anti-infection treatment after admission, and the degree of immunosuppression was reduced. Antibiotics were adjusted in time according to the results of mNGS or urine culture and DST (Table 5). In culture-negative cases, the targeted adjustment rate of antibiotic treatment was 76.9% (10/13), according to mNGS, whereas, in culture-positive cases, the rate was 33.3% (2/6), indicating no significant difference (*p* = 0.129). Meanwhile, 69.2% (9/13) of the solely mNGS-positive cases and 66.7% (4/6) of culture-positive cases showed no RUTI at 6 months of follow-up (no significant difference, *p* > 0.999). The cure rate was 68.4% (13/19).

**Table 5:** Adjustment of antibiotic treatment according to mNGS results.

**Patient**	**Antibiotic treatment before testing**	**Adjusted antibiotic treatment**
P1	Levofloxacin	Amoxicillin–clavulanic acid
P2	Levofloxacin + amikacin	Fluconazole
P3	Biapenem	Fluconazole + fosfomycin
P4	Levofloxacin + amikacin	Amoxicillin–clavulanic acid
P5	Ceftizoxime	Fosfomycin + ganciclovir
P6	Ceftizoxime	Fosfomycin
P9	Biapenem + ganciclovir	Nitrofurantoin + fluconazole
P10	Biapenem	Amoxicillin–clavulanic acid + ganciclovir + fluconazole
P11	Levofloxacin	Levofloxacin + ganciclovir + fluconazole
P12	Biapenem	Biapenem + ganciclovir
P14	Levofloxacin + linezolid	Fluconazole
P16	Moxifloxacin + biapenem	Amikacin

## Discussion

For KTRs, urinary tract infections present recurrent and refractory characteristics because of the long-term application of immunosuppressants, the anatomical changes of the urinary system, and the repeated dialysis before transplant, which may result in impaired kidney function, and graft failure, or even death (3, 4). However, the current clinical detection of RUTI pathogenic microorganisms relies mainly on urine culture, and the negative rate has been reported to be up to 80% (13). The low accuracy and long wait for urine culture results drive the empiric use of antimicrobial therapy for treating RUTI after transplant. Mismatches between anti-infective therapy and susceptibility are inevitably more frequent with empiric therapy, diminishing the protective flora and increasing costs, resistance, and treatment failure. These considerations highlight the need for rapid and accurate pathogens detection and identification methods.

Fortunately, mNGS technology, with the characteristics of fast detection speed, high sensitivity, and broad coverage, can effectively compensate for the deficiency of urine culture and offer a very significant practical advantage for RUTI after transplant. It directly extracts all the nucleic acid fragments in the specimen, sequences them, and compares the reference sequences in the microbiology-specific database with the specimen sequences. It then analyzes the sequences by intelligent algorithms to obtain the number of microorganisms in the specimen that have the same sequence number as various reference pathogenic microorganisms, which can avoid the missed detection of difficult-to-culture pathogenic microorganisms (14–17). As the disease progresses and after repeated antibiotic treatment, the dynamics of microbial species in urine samples can be clearly identified by mNGS. Antibacterial drugs have fewer impacts on mNGS than conventional cultures, and the treatment strategies can be adjusted according to the test results. Currently, the most reported tests were mNGS on blood, alveolar lavage fluid, and cerebrospinal fluid, which can rapidly and accurately identify microbial species, improve clinical diagnosis, and guide effective clinical treatment. However, due to the complex microbial background of urine samples, the application of mNGS in infections of the urinary and reproductive systems is still in the verification stage (18). Li et al. (9) reported a case of an unexplained febrile patient with negative clinical culture results, and after the final mNGS of blood and urine, the cause of the disease was clearly identified as *Enterococcus faecalis* infection in the urine, which was quickly controlled with the appropriate antibiotics. Mouraviev and Mcdonald (11) pointed out that NGS has a high sensitivity in diagnosing urinary tract infections and can be used to develop a precise treatment based on the results. Dixon et al. (19) published a review article that explored the limitations of traditional culture methods and demonstrated the progress of research across the urinary microbiome, the pathogen spectrum, and the clinical applications in several areas of urology. These studies were conducted on general urinary tract infections. However, RUTI in KTRs, due to unique features of urological anatomy and particular immunosuppressive state, may have different characteristics and needs further research. Therefore, we performed mNGS in 19 KTRs with RUTI and obtained microbiological profiles of urine samples from these patients to provide the basis for clinical treatment.

All urine samples were tested by mNGS, and bacteria were detected in 17 cases, with a higher positive rate of 89.5% than 31.6% in urine culture (*p* < 0.05). We found that the urinary tract in KTRs with RUTI harbored a rich and complex microbiota by mNGS, in which the dominant groups of bacteria were *Escherichia coli, Klebsiella pneumoniae, Staphylococcus epidermidis, Micrococcus luteus, Lactobacillus iners*, and *Enterococcus*. Previous studies showed that *Escherichia coli, Klebsiella pneumoniae, Proteus mirabilis, Enterococcus*, and *Staphylococcus putrefaciens* were the most common pathogenic microorganisms in general urinary tract infections (20–22), which were slightly different from our findings. These outcomes suggest that RUTI in KTRs is attributable not only to relapse with the original infecting strain but also to reinfection with a new strain. Such a clear microbiota facilitates the overall control of empirical treatment by transplant surgeons to ensure the coverage of all possible bacterial pathogens and resistance profiles. Four bacteria were detected in six positive urine culture cases, which are consistent with the positive rate reported in the previous literature (14). Thirteen cases failed to detect pathogenic bacteria, but mNGS made up for this deficiency by detecting the presence of potentially pathogenic bacteria in 11 of them. Moreover, in the other two cases, although no pathogenic bacteria were detected, fungi were detected, allowing the patient's diagnosis to be clarified and the treatment to be redirected in time.

Since urine culture cannot detect viruses, the advantage of mNGS for virus detection in urine is highlighted. Mai et al. (23) reported a 16-year-old encephalitis boy with all clinical cultures negative, but eventually, a rare *flavivirus* was detected by urine mNGS, which led to the diagnosis of *Japanese encephalitis virus* and provided a direction for the treatment. Our findings unexpectedly showed that viral nucleic acid fragments were detected in all urine samples, but the number of nucleic acid fragments was more than 100 in 11 cases, with a positive rate of 57.9%. These included *JC polyomavirus*, CMV, *BK polyomavirus, human herpesvirus type 6A*, and *human alphaherpesvirus 1*, which could probably affect the function of the transplanted kidney. Notably, the TTV ranked only after *JC polyomavirus* and CMV in this study, expanding our imagination. Although the most common virus infecting the urinary tract of KTRs is *BK polyomavirus*, which is also a significant cause of renal allograft injury and failure (24–26), and only one person was *BK polyomavirus*-positive in this study. Thus, we have reasons to believe that the low positive rate of *BK polyomavirus* in KTRs with RUTI may be due to the reduction of immunosuppression in consideration of RUTI. Herpesvirus infections in KTRs, especially CMV infections, have been relatively well-understood (25, 27, 28), and *JC polyomavirus* infections have developed progressively (29, 30). It is noteworthy that the association between TTV and the immune system of KTRs is somewhat unclear to date (31–34). However, TTV DNA is emerging from an inconsequential viral pathogen to a marker of immune function with the potential to predict infection and rejection (34). At the same time, whether RUTI symptoms cause any changes in TTV infection in KTRs or whether they contribute to the manifestation of RUTI symptoms is unclear and merits further in-depth investigation. On the one hand, this study paves the way for the next step in studying the relationship between the individual risk of RUTI and immunity to KTRs; on the other hand, it provides the basis for personalized anti-infective strategies. The development of RUTI may not be solely a matter of bacterial infection, and viruses may also play an essential role in pathogenesis. The possible co-infection with bacteria and viruses of RUTI needs to be taken into account by transplant surgeons. However, there are few detailed studies on bacterial and viral co-infection in KTRs with RUTI, which we need to work on in the next step.

Eight cases of fungal infections were detected in these samples by mNGS, namely *Malassezia restricta, Aspergillus flavus, Malassezia globosa, Candida albicans, Meyerozyma guilliermondii*, and *Fusarium graminearum*. *Malassezia*, a conditionally pathogenic fungus, mainly parasitizes superficial areas of human sebaceous glands and causes folliculitis, such as the chest, back, head, face, and neck. It can also cause disease in KTRs reportedly (35). The detection of it cannot necessarily be considered contamination. *Aspergillus* and *Candida albicans* are fungal infections frequently seen in immunosuppressed patients. Cases 2 and 14 had fevers without the identified causes. Urine mNGS suggested *Candida albicans* and *Meyerozyma guilliermondii* infections, which were effectively cured by antifungal treatment, demonstrating the great value of mNGS in clinical diagnosis and guiding treatment. However, none of the urine cultures cultured fungi in this study.

Apart from this, we also found that every patient after transplant diagnosed with RUTI could carry a variety of pathogens in the urethra. mNGS showed a significantly higher proportion of poly-microbial infections identified than culture (100.0 vs. 10.5%; *p* < 0.05). The identification of infections of multiple pathogens can guide effective anti-infection treatment. Additionally, the information on antibiotic resistance provided by mNGS from urine samples of KTRs with RUTI can help evaluate the infection risk. We analyzed the antibiotic resistance genes of the bacteria detected in the mNGS test. Five patients detected three drug-resistant bacteria, including *Klebsiella pneumoniae, Escherichia coli*, and *Enterococcus faecalis*, suggesting that these three bacteria are common drug-resistant bacteria in KTRs with RUTI. Among them, *Klebsiella pneumoniae* was detected with various drug-resistant genes, which brought difficulties to the treatment. Nevertheless, we achieved an excellent curative effect after the adjustment of antibiotics in time according to mNGS, demonstrating the advantages of mNGS in treating KTRs with RUTI.

Though it costs moderately, urine culture is laborious and has to test repeatedly when the previous test results are negative, which is time consuming. Nevertheless, mNGS has a shorter turnaround time, and the cost will continue to decline, despite the current cost of mNGS being higher than that of conventional testing. Fortunately, the cost is affordable for KTRs. Moreover, mNGS can lead to a reduced length of stay and cost savings in the healthcare system through timely recognition and treatment, especially for the immunocompromised KTRs, in whom the spectrum of potential pathogens is greater and incurs substantial medical costs associated with anti-infective therapy and the resultant risks of rejection. The difference in cost-effectiveness needs to be interpreted with caution since no patient in this study was tested as a control for the comparison of medical costs.

Despite the great value of mNGS in infectious diseases, there are still many practical problems in its clinical application. More than 99% of the reads generated by sample sequencing are from human hosts (36), while microorganisms represent only a small percentage. Sequencing all nucleic acids reduce the sensitivity of pathogen identification, making it difficult to distinguish between colonizing, background, and pathogenic microorganisms among the various species detected (37). However, it is possible to deplete host nucleic acids by certain methods (38, 39), and reducing the human-derived nucleic acid sequence proportion can increase the amount of microbial data and improve sensitivity to some extent. In addition, the microbiota of RUTI in KTRs detected in this study not only is limited to pathogens but also may contain health-associated commensal bacteria. In any case, determining mNGS results requires a combination of nucleic acid fragment counts, clinical presentation, and other laboratory results. Another limitation is that the number of patients included in this study was small because the incidence rate of RUTI in our renal transplant center is relatively low, which may attribute to our significant attention to the first episode of infection and meticulous management. So the sample size was mainly based on practical considerations. We hope to conduct a multicenter study to increase the sample size in the future.

In conclusion, our study demonstrates the potential application of mNGS in detecting urinary pathogenic microorganisms in KTRs with RUTI, especially in samples that are negative for conventional culture. This study also expands our insights into the pathogens of RUTI in KTRs, enabling further research and providing new diagnostic and treatment options for RUTI in KTRs. As the cost of mNGS decreases, while the precision improves, KTRs with RUTI would be expected to benefit more from it.

## Data availability statement

The datasets presented in this study can be found in online repositories. The names of the repository/repositories and accession number(s) can be found at: https://www.ebi.ac.uk/, PRJEB52041.

## Ethics statement

The study involving human participants was reviewed and approved by the Clinical Research Ethics Committee of Henan Provincial People's Hospital. The patients/participants provided their written informed consent to participate in this study. Written informed consent was obtained from the individual(s) for the publication of any potentially identifiable images or data included in this article.

## Author contributions

WD and XT contributed to the study conception, design, and drafting of the manuscript. WD, YY, and JZ contributed to the acquisition, analysis, interpretation of data, and performed statistical analyses. XT and TY provided administrative, technical, material support, and study supervision. All authors contributed to the article and approved the submitted version.

## Funding

This work was supported by the Project of Science and Technology of Henan Province (Nos. 202102310438 and 222102310264), the 23456 Talent Project Foundation of Henan Provincial People's Hospital (No. ZC20200327), the Joint Construction Project of Henan Medical Science and Technology Research Plan (Nos. LHGJ20210042 and LHGJ20210068), and the Foundation of Henan Educational Committee (No. 22A320012).

## Conflict of interest

The authors declare that the research was conducted in the absence of any commercial or financial relationships that could be construed as a potential conflict of interest.

## Publisher's note

All claims expressed in this article are solely those of the authors and do not necessarily represent those of their affiliated organizations, or those of the publisher, the editors and the reviewers. Any product that may be evaluated in this article, or claim that may be made by its manufacturer, is not guaranteed or endorsed by the publisher.
